# Effect of different recruiter attire on trial participation in Lesotho: a randomized study within a trial

**DOI:** 10.1186/s13063-026-09638-y

**Published:** 2026-03-20

**Authors:** Grace H. Yoon, Sandro Stoffel, Soheila Aghlmandi, Ntotiseng Lesaoana, Palesa Mahlatsi, Makobefo Chakela, Jennifer M. Belus, Matthias Briel

**Affiliations:** 1 Division of Clinical Epidemiology, Department of Clinical Research, University Hospital Basel, University of Basel, Totengässlein 3, Basel, 4051 Switzerland; 2 Epidemiology, Biostatistics, Prevention Institute, University of Zurich, Hirschengraben 84, Zurich, 8001 Switzerland; 3 Health Economics Facility, University of Basel, Klingelbergstrasse 61, Basel, 4056 Switzerland; 4 Research Department of Behavioural Science and Health, University College London, 1–19 Torrington Place, London, WC1E 7HB UK; 5 Institute of Pharmaceutical Medicine (ECPM), University of Basel, Klingelbergstrasse 61, Basel, 4056 Switzerland; 6 Pediatric Research Center, University Children’s Hospital Basel, Spitalstrasse 33, Basel, 4031 Switzerland; 7 SolidarMed Lesotho, Main North 1 Road, Butha Buthe, Lesotho; 8 Department of Psychology, University of Zurich, Binzmühlestrasse 14, Zurich, 8050 Switzerland

**Keywords:** Recruitment, Participation bias, Attire bias, Expectation state, Study within a trial, Embedded randomized controlled trial

## Abstract

**Background:**

Effective participant recruitment is central to generating evidence in randomized controlled trials. Clinical trials have increased in Lesotho, but research processes are rarely assessed in this setting. The recruiter’s attire is a modifiable factor that may influence a patient’s decision to participate in a trial upon being approached. In healthcare domains, white coats convey clinical authority over patients seeking help for their health problems. This study aimed to determine the effect of clinical trial recruiter attire on trial participation rates in a peri-urban district outpatient clinic in Lesotho.

**Methods:**

A cluster-randomized study within a trial (SWAT) was conducted with two recruiters, who were daily randomized to wear white coats or casual attire. The SWAT was embedded in a vignette-based trial testing the impact of social endorsement of a new treatment on patients’ uncertainty attitudes, and its data source was the host trial’s prescreening logs. The primary outcome of the SWAT was the participation rate, defined as the rate of patients who accepted participation in the host trial out of those approached. Negative binomial mixed effect regression was used to model attire and recruiter effects on participation with day-based random effects.

**Results:**

Overall, 70% of patients accepted participation in the host trial out of 682 approached for recruitment. Among these, 71% of 374 patients approached by a recruiter wearing white coats and 68% of 308 patients approached by a recruiter in casual attire accepted trial participation (adjusted incidence rate ratio [aIRR], 1.03; 95% confidence interval [CI], 0.86–1.24; ref = casual). The older male recruiter yielded lower participation compared to the younger female recruiter (aIRR = 0.86; 95% CI = 0.69–0.98). No interaction effects were observed between attire conditions and recruiters.

**Conclusions:**

We found no evidence that attire is associated with improved participation rates in Lesotho. Further investigations on other recruiter characteristics alongside trials may identify meaningful targets to improve clinical trial recruitment in this setting.

**Trial registration:**

The host trial and its SWAT protocols were preregistered in OSF.io (N6xc2) and registered in ClinicalTrials.gov (NCT06246058). February 07 2024.

**Supplementary Information:**

The online version contains supplementary material available at 10.1186/s13063-026-09638-y.

## Background

Effective participant recruitment is central to generating evidence in randomized controlled trials. “Study within a trial” (SWAT) is a study design that allows for the examination of research processes alongside a host trial [1, 2]. SWATs are gaining traction for their resource efficiency in answering important meta-research questions alongside primary clinical questions without significant additional costs, adding contextual and pragmatic evidence on how to conduct research more effectively. While SWAT evidence on how to optimize clinical trial recruitment is concentrated in high-income countries [3], the resource and ethical implications of generating evidence for improving research practices are arguably higher in low- and middle-income countries (LMICs). Not only are research resources scarce in LMICs, but research in these settings are often focused on vulnerable, at-risk populations. However, little is known about modifiable factors that contribute to successful recruitment of patients for randomized clinical trials in low-resource settings where such research activities are rising [4].

Expectation theory states that socially built expectations for individual characteristics impact how individuals interact with others [5, 6]. Appearances commonly attributed to specific social roles, such as a woman with an apron to a homemaker, may lead to biases on how individuals respond to these external characteristics based on their assumptions tied to these roles, such as an assumption that a younger female homemaker may have less decision-making power in the household than an older male breadwinner. As exemplified in the analogy of the homemaker, appearance-based biases may arise from gender [7, 8], age [9], and attire [10, 11]. Biases arising from these appearance-based characteristics have mostly been studied in the field of business in the context of recruitment or gender studies in the context of power relations. These concepts of appearance-based biases have also been applied in health service contexts in exploration of determinants of patient-provider relationships, as relational factors like trust are consequential for the patients’ likelihood to seek care for emerging symptoms and for preventative care [12–14]. For example, higher perceived attractiveness of physicians was negatively associated with patients’ perception of their professionalism [8]. In the same study, sex-concordance between the patient and provider (i.e., female patient with a female provider) contributed to this negative bias [8], contradicting the common assumption that demographic concordance improves trust between two parties. These biological characteristics like perceived attractiveness and sex-concordance may contribute to how patients respond to clinical research recruiters, but they are not easily modifiable factors given limited resources.

Unlike biological factors, attire is easily modifiable and may influence patients’ decisions in clinical settings, such as their decision to uptake prevention and treatment programs proposed by their provider. Biases attributed to attire have been established in broader sociological contexts, such as females receiving more positive responses after interviews when wearing masculine clothing versus feminine clothing [11]. Cognitive biases, such as the assumption of authority, trustworthiness, and superiority, may arise from the perceiving of widely established characteristics of a clinical provider, for both providers [15] and patients [16]. In clinical settings, prior evidence points to a provider’s white coat attire being associated with higher levels of perceived authority by the patient, contributing to the provider’s trustworthiness [16, 17]. Within the realm of clinical research, one study from two decades ago in a high-resource, inpatient setting found a null effect of attire in research participation rates [18], but this evidence cannot be generalized to other settings. This question remains unanswered in different sociocultural and regional contexts, such as in Southern Africa and LMICs, where clinical trials are expanding. A recent systematic review demonstrated that the current evidence base of SWATs originates solely from high-income settings and places a disproportionate focus on patient information and incentive models as exposures and study retention as the outcome of interest [3]. Therefore, there is a dearth of SWAT evidence on the influence of altering recruiter characteristics on participant recruitment, let alone other study administrative factors. One study expounded on the role of open versus blinded study design as a significant contributor to recruitment rates [19], but did not examine any other administrative factors related to recruitment. As the evidence base related to recruiter attire in clinical trials is dated and not generalizable to global research contexts, the current study attempts to close this evidence gap at an outpatient clinical trial recruitment context in Lesotho, an LMIC situated in Southern Africa.

While the perceived role of white coats in clinical contexts is shifting among providers [20, 21], an updated systematic review in 2025 confirms that patient perceptions of provider trustworthiness and professionalism are still significantly impacted by the provider’s attire [22]. Likewise, insights from local partners in Lesotho indicated that service users of the district hospital predominantly associate white coat attire with clinical and knowledge authority. Therefore, when an unknown research recruiter who is not affiliated with clinical services proposes a new trial to a patient as a potential participant, the patient may exhibit different attitudes to research participation when the recruiter is wearing a white coat versus not wearing a white coat. This study aimed to determine the effect of clinical trial recruiter attire on trial participation rates at an outpatient clinic in a government district hospital located in a peri-urban center in Butha Buthe district, Lesotho. Based on prior evidence in clinical contexts where patients reported higher satisfaction with providers wearing white coats [16, 23], we hypothesized that recruitment conducted in white coat attire would yield higher participation rates.

## Methods

The reporting of this study follows the SWAT reporting guidelines from Trial Forge Guidance [24].

### Study design

We conducted a cluster-randomized SWAT with two recruiters who were daily randomized to wear a white coat versus casual attire on patient participation rates in a vignette-based host trial.

### Setting

In Lesotho, clinical research activities have been growing since the early 2000s. The most established and recognized research efforts include national health surveillance surveys and long-term prevention campaigns for human immunodeficiency virus (HIV) and tuberculosis conducted by multilateral, non-governmental organizations (e.g., World Health Organization, United Nations Development Program), in partnership with the Ministry of Health. Clinical research specifically in Butha Buthe district emerged in the 2010 s, concentrated in the district hospital and select health facilities near remote villages to improve disease detection and treatment outcomes in the population, led primarily by international non-profit organizations and academic entities in collaboration with the district health management team.

Today, patients using public health services in Butha Buthe may be moderately aware of research and many may be able to differentiate between research activities and actual health services. While some patients who are familiar with research remuneration may react positively to potential financial gains associated with trial participation, others may be hopeful of downstream benefits of research projects, such as improved health services and outcomes for the community. Those who are wary of clinical trials in Lesotho may have negative attitudes toward engaging with external (i.e., foreign) influences due to prior encounters with external healthcare providers and researchers, and/or exposure to negative local rhetoric regarding specific external bodies in their community. These potential sources of negative attitudes align with known barriers of trial participation in other settings [25]. Clinical trial recruitment in Lesotho is generally conducted by existing health providers, project-based research staff hired by the implementing organization, or by community members recruited as village or community health workers.

For the current SWAT, recruitment activities took place in the waiting room of the outpatient department at the Dr. Strong Makenete Hospital (formerly Butha Buthe Government Hospital), a district hospital with a catchment area of approximately 122 thousand residents [26]. Data collection for this study was conducted by trained non-clinical research staff from the implementing organization to minimize the impact on existing workflows and staff at this busy district hospital.

### Host trial

The SWAT was embedded in a vignette-based behavioral survey trial testing the effect of social endorsement on uncertainty attitudes toward an ambiguous, hypothetical medical treatment. The primary outcome of the parent study was the total score from the Ambiguity Aversion regarding Medical Tests and Treatment measure [27]. Recruiters informed participants that participation in the host trial would involve a one-time, interviewer-led survey lasting 15–30 min before or after their doctor’s visit. No identifiable information was collected, and no biomedical procedures were performed as part of the host trial. The protocol for the SWAT and the host trial can be found at OSF.io (N6xc2) and both were approved by the Research Ethics Committee National Health Research Ethics Committee of Lesotho (NHREC ID 213–2023).

### Participants and procedures

The host trial’s inclusion criteria were patients aged 18 years and older, who were literate in Sesotho and available for 30 min before or after their clinical visit at the hospital. The host trial evaluated patients’ health attitudes toward a hypothetical new treatment for their health issue using a low-burden vignette-based survey, which did not involve any biomedical procedures or the collection of identifiable information. Participants were recruited from the waiting area of the outpatient clinic. Individuals present in this area who did not have a health issue (i.e., accompanying their loved ones, collecting documentation) were excluded from the trial. Data from face-to-face recruitment were collected and entered manually on daily prescreening logs by study recruiters. Prescreening logs were used to record the number of participants approached, reasons for non-participation, and other comments on the recruitment process. Visit reasons were recorded for all enrolled participants. No identifiable or demographic data were recorded on prescreening logs, therefore not collected for the SWAT.

### Intervention

**Fig. 1:**
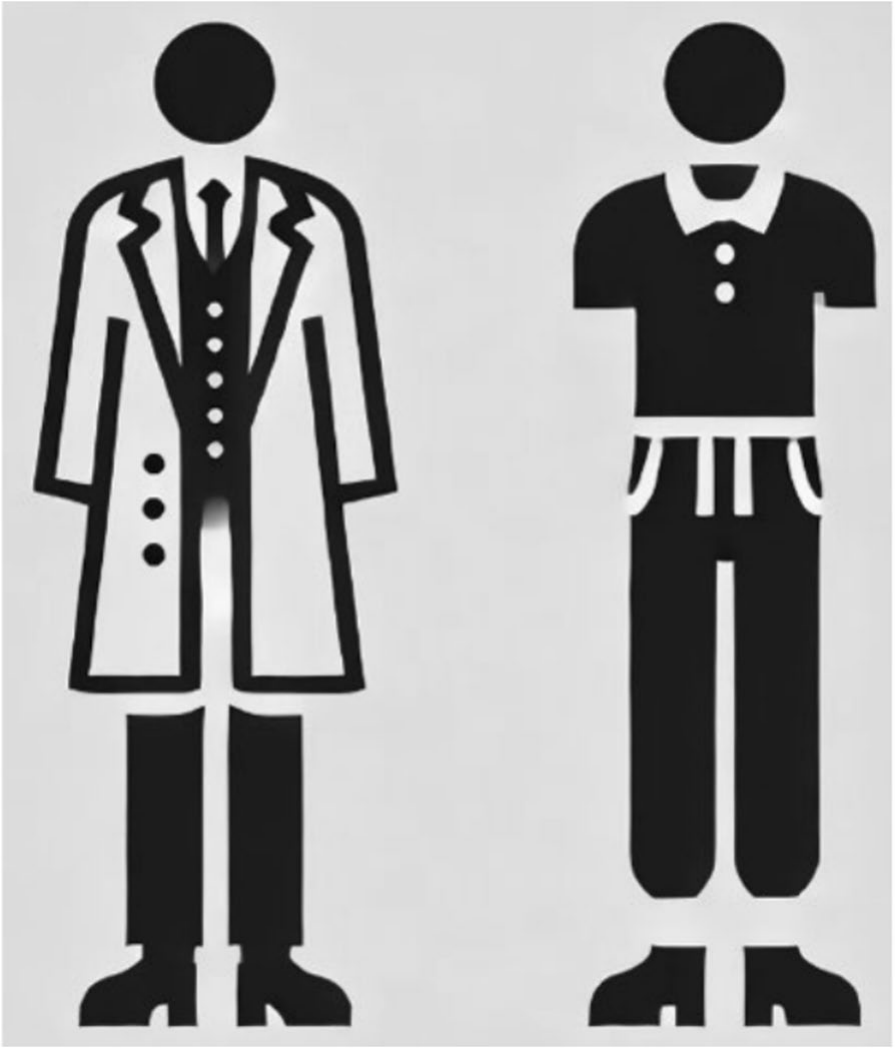
Visual representation of the two attire conditions

Recruiters were randomized to wear a white coat or no white coat on top of business casual personal wear. Recruiters presented to the recruitment site wearing the assigned attire and conducted recruitment for the host trial from March to September 2024. Figure 1 illustrates the basic components of the two attire conditions.

### Outcomes

The primary outcome of the SWAT was the participation rate, defined as the rate of patients who accepted participation in the host trial out of those approached.

### Sample size

No formal sample size calculation was performed for the SWAT, as the SWAT sample size was dependent on the host trial. There were no interim analyses or stopping rules planned for the SWAT. For the host trial, we determined the sample size based on a priori effect size *d* = 0.3 averaged from a meta-analysis on the effect of social support on health attitudes [28], with 1:1 allocation between two survey arms. With 400 respondents included in the host trial’s per protocol analysis, we achieved a statistical power of 0.85.

### Randomization and data collection

Random allocation for the two attire conditions occurred at the day level. Survey day clusters were assigned to either casual or white coat attire using simple random sampling, without stratification or blocking. A “day cluster” refers to the cluster of participants who were recruited on the same calendar day per recruiter. Consecutive numbers representing each survey day (1–61) were randomized to one of two groups using Microsoft Excel and saved onto an encrypted server hosted by the University Hospital Basel. Random allocation sequence generation, communication of day-based attire assignment, and allocation concealment were performed by the principal investigator who had no influence on the day-to-day data collection activities of the recruiters and was primarily based overseas. The SWAT’s randomization scheme was developed and implemented separately from the randomization of the host trial, which randomized participants to one of two survey vignette conditions at the individual level.

Two local recruiters (i.e., of Basotho ethnicity, speaking Sesotho) enrolled participants for the host trial and collected prescreening data for the SWAT. One recruiter was a female social worker in her early-30s, and another was a male pastoral counselor in his mid-40s. The female recruiter and male recruiter were randomly assigned to both conditions (casual or white coat) at the same rate throughout the trial. The two recruiters recruited participants at the outpatient clinic on different days from each other. Only one attire condition was assigned per day (i.e., treatment group—white coat attire, control group—casual attire). There were no days when both white coat and casual attire were implemented in the outpatient waiting area on the same day to mitigate the risk of contamination (i.e., a potential participant may observe another individual being approached in a different attire than their own recruiter). On a weekly basis, survey days were assigned an attire condition (i.e., day 8—casual), but recruiters carried out these assigned days on different days of the week according to their availabilities (i.e., female recruiter’s day 8 may be completed on Monday and the male recruiter’s day 8 may be completed on Wednesday of a given week). Specific days of the week and hours of the day when recruitment occurred (i.e., morning versus afternoon) were not randomized. Of note, most recruitment activities took place during the early morning hours as the outpatient waiting area is most busy during this time of the day.

Recruiter adherence was monitored via daily communication with the recruiters. The principal investigator, who randomized the recruitment days to either white coat or casual attire, communicated this assignment to the recruiters one working day before the assigned day. The recruiters confirmed receipt of this message and their adherence to the assigned attire (i.e., confirmation that they had prepared and brought the white coat) before the start of the day. On most occasions, white coats were left at the recruiter’s office unless they had to be taken for washing to allow for use when the assignment required them. Although the SWAT did not monitor the base attire that recruiters wore under the white coat and how recruiters dressed as casual attire, recruiters dressed in work-appropriate clothing in compliance with the implementing organization’s standards for professional conduct.

Two female data officers, based in the study office, transferred the manual data to an electronic master dataset and performed quality control checks on the manually entered source data. All data entered in the master dataset were double checked with the source data by the principal investigator. One female local study representative also assisted with data entry and liaised between hospital administration and the study team in the absence of the principal investigator. All supporting study members, who facilitated the conceptualization and implementation of the SWAT jointly with the principal investigator, were Basotho individuals living in the district who were familiar with the local healthcare environment.

### Analysis

Descriptive analyses were conducted to show the count of approached patients, enrolled participants, reasons for non-participation, and sample characteristics of enrolled participants (from host trial data) into tables. For statistical analysis, we fit a negative binomial mixed effect model with the count of acceptances (i.e., agreement to participate in the host trial) as the dependent variable and attire condition as the independent variable, offset by the count of approached patients, adjusting for day-based random effects and recruiter effects. The negative binomial model was selected for its reliability and appropriateness for our count outcome data (i.e., acceptances). As the outcome data exhibited overdispersion in preliminary examinations of its distribution, Poisson or linear models would not have been appropriate for this analysis. We calculated adjusted incident rate ratios (aIRRs) accompanied by 95% confidence intervals (CIs). Results were evaluated at an alpha level of 0.05. We also examined interaction effects of attire and recruiter. For sensitivity, we examined variations in participation and approached rates over time. Analysis was conducted in R version 2024.07 [29].

## Results

### Participant flow

**Fig. 2:**
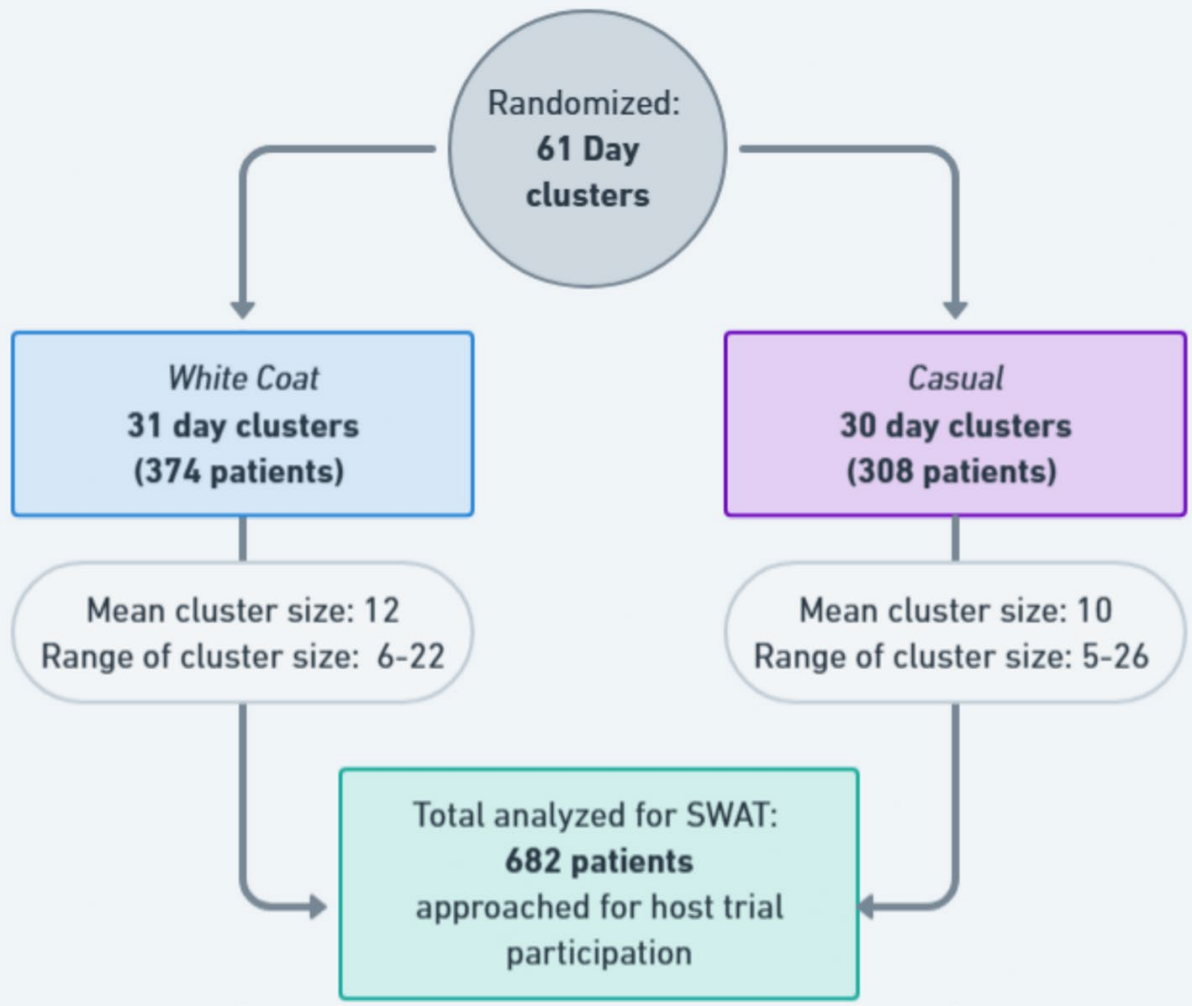
Participant flowchart

Overall, 682 patients were approached as part of recruitment for the host trial, of which 70% of patients were successfully recruited for the host trial. Approached patients were cluster-randomized on 61 days for the recruiter’s attire assignment. The period of the SWAT was the same as its host trial, which ran from March to September 2024. Thirty-one days with 374 patients were approached by recruiters in white coat attire, averaging 12 (range 5–20) patients per day cluster; 30 days with 308 patients were approached by recruiters in casual attire, averaging 10 (range 5–26) patients per casual day cluster. The participant flowchart is shown in Fig. 2.

### Participation rates

Stratified descriptions of the effect of attire and recruiter conditions on participation rates are summarized in Table 1. Overall, 70% of patients accepted participation in the host trial out of 682 approached for recruitment. By raw proportions, recruitment in white coat attire yielded higher participation rates by 3 percentage points (71% vs. 68%), and recruitment by the female social worker yielded higher participation by 14 percentage points (77% vs. 63%). Mixed model results revealed no significant differences in participation rates based on attire (aIRR, 1.03; 95% CI, 0.86–1.24). The male recruiter yielded lower participation among patients at the outpatient clinic (aIRR, 0.82; 95% CI, 0.69–0.98). There were no significant interaction effects between the attire condition and recruiter assignment (Appendix 1).

**Table 1:** Descriptive and statistical results

Total patients approached, *N* = 682	Patients approached, *N*	Patients recruited, *N* (%)	Adjusted incidence rate ratio^a^ [95% confidence interval]
**Attire**			
White coat	374	264 (71%)	1.03 [0.86, 1.24]
Casual	308	210 (68%)	Ref
**Recruiter**			
Female social worker (early-30s)	310	239 (77%)	Ref
Male pastoral counselor (early-40s)	372	235 (63%)	0.82 [0.69, 0.98]

^a^Negative binomial mixed effect model with two independent variables (attire, recruiter) and day-based random effects

The SWAT generated minimal costs in addition to the host trial, which included the procurement of white coats and the study team’s time to prepare and work on the project. Preparatory activities included hours for building and translating materials and rehearsing in-person recruitment and prescreening procedures. Salary costs of the three data officers and two recruiters who worked on these tasks in Lesotho were accounted for at approximately 0.05 FTE (~2 h per week) over 1 month prior to recruitment. The costs are listed in Table 2.

**Table 2:** SWAT costs

Currency^a^	White coats (×2)	Preparation	Total cost	Per day	Per patient
Lesotho Loti	470	4444.63	4915.62	80.57	7.51
US Dollars	26.63	251.82	278.51	4.56	0.43

^a^Conversion rate is from September 2024; 17.65 LSL = 1 USD [30]. *LSL*, Lesotho loti, *USD*, US Dollars

#### Characteristics of recruited patients

Prescreening data used for the SWAT did not include any sociodemographic data, but characteristics of individuals who accepted participation were available to link from the host trial. There were no relevant differences in patient characteristics between the two randomized attire groups (Table 3).

**Table 3:** Characteristics of host trial participants by recruiter’s attire condition

Characteristics	Total patients (*N* = 474)	Recruited in white coat (*N* = 265)	Recruited in casual attire (*N* = 209)
Age, median (IQR)	38 (28–47)	37 (27–46)	38 (29–49)
Sex at birth (female), *N* (%)	360 (76)	205 (77)	155 (74)
Education^a^, *N* (%)			
Primary or less	89 (19)	49 (19)	40 (19)
Secondary	245 (52)	134 (51)	111 (53)
Tertiary	139 (29)	81 (31)	58 (28)
Employment^a^, *N* (%)			
Full-time/self-employed	112 (24)	61 (23)	51 (24)
Part-time	45 (9.5)	21 (8)	24 (11)
Unemployed	315 (67)	181 (69)	134 (64)
Monthly income in Lesotho Loti^b^, median (IQR)	850 (0–3000)	700 (0–3000)	950 (0–3000)
Travel time (minutes), median (IQR)	30 (20–60)	30 (20–60)	30 (25–60)
Visit reasons^c^, *N* (%)			
Cardiac	95 (22)	55 (25)	40 (19)
Endocrinological	67 (16)	32 (14)	35 (17)
Dermatological	39 (9)	26 (12)	13 (6)
Dental	35 (8)	18 (8)	17 (8)
Psychiatric	39 (9)	22 (10)	17 (8)
Orthopedic	32 (7)	19 (8)	13 (6)
Reproductive	22 (5)	13 (6)	9 (4)
HIV/other STIs	46 (11)	13 (6)	33 (16)
Other	57 (13)	26 (12)	31 (15)

*Abbreviations*: *IQR— *interquartile range, *STI—*sexually transmitted infection

^a^Unknown = 1 in education; = 2 in employment

^b^Currency reported is Lesotho loti (17.65 LSL = 1 USD, September 2024 [30]). Excluding 60 observations which were refused or missing, total observations = 415 (casual = 186; white coat = 229)

^c^Response options were not mutually exclusive. Options with cell counts below nine were aggregated as “Other”– oncology, ophthalmology, accidental or violent injuries, ear-nose-throat, gastroenterology, respiratory, urology, and hematology.

#### Reasons for non-participation

Potentially eligible patients who rejected the host trial were given the opportunity to provide a reason for non-participation by the recruiter. Patients were presented with possible reasons and could select all that apply. These responses were counted and summarized in Table 4, by attire and by recruiter. The most common reason for non-participation was lack of interest (43% of all reasons given), whereas only 9% reported illiteracy or being otherwise visually or hearing impaired, impacting their ability to read and understand survey instructions. Descriptively, lack of interest was cited as a reason for refusal to participate primarily when recruiters were dressed in casual attire, whereas more patients cited time constraint as a reason for non-participation when recruiters were dressed in white coat attire. This tally excludes those who were ineligible by age (i.e., under 18 years of age, *n* = 11) or visit reason (i.e., not visiting as a patient, *n* = 96).

**Table 4:** Reasons for non-participation

Reason	Total	White coat	Casual	Female recruiter	Male recruiter
No interest, *N* (%)	41 (43)	15 (37)	26 (63)	6 (15)	35 (85)
Time constraint, *N* (%)	33 (35)	23 (70)	10 (30)	11 (33)	22 (67)
Illiteracy or diminished vision/hearing, *N* (%)	9 (9)	5 (56)	4 (44)	5 (56)	4 (44)
Need urgent care, *N* (%)	12 (13)	6 (50)	6 (50)	7 (58)	5 (42)

#### Sensitivity analyses

There were no visible differences in the number of patients approached or the participation rate over time during the recruitment period of the host trial by attire or recruiter (Appendix 2).

#### Harms

There were no harms or unintended effects collected during the SWAT.

## Discussion

Current evidence is limited on easily modifiable study-side factors to improve clinical trial recruitment. We tested the effect of white coat attire versus casual attire on trial participation rates for a vignette-based randomized controlled trial in Lesotho. Recruiter attire did not yield significant differences in the participation rate. There were also no statistically significant differences between the two individual recruiters in the participation rate.

### Contextualization of results in broader literature and within the local context

Consistent with prior evidence from a high-resource inpatient setting in the USA [18], the current SWAT did not find a significant association between white coat attire and clinical trial participation rates in Lesotho. Given our existing knowledge on appearance-based cognitive biases in health contexts, we hypothesized that the white coat attire, a visible marker for clinical authority, worn by researchers, may trigger differences in the rate of trial participation among patients attending a busy outpatient clinic [6]. However, our findings did not support this hypothesis. The lack of effect of white coat attire on trial participation rates may be explained by prior qualitative evidence from Ghana and South Africa, where clinical trial participants expressed that their perceived relationship with researchers was different in nature than their relationship with clinical providers, stating that they see research participation as more of a *partnership* rather than a *patient-doctor* dichotomy [31]. This indicates that the behavioral influence of appearance-based biases placed on providers may not directly apply to recruiters for clinical trials.

Further, evidence on white coat preferences may be waning due to white coats becoming less ubiquitous as a symbol for clinical authority among patients [20], especially as younger physicians opt out of wearing white coats to lessen appearance-based power dynamics between them and their patients [32]. Finally, preferences elicited from 1506 primary care patients in Belgium indicate that patient preferences on physician attire differ based on the physician’s demographics. Among these patients, older male and female physicians were preferred with white coats, while younger physicians were preferred without white coats [10]. This evidence indicates that cognitive biases from specific attire conditions may not produce the same effect across recruiters of different age and sex groups, which may also explain the results in the current study with one male and one female recruiter.

Research in Lesotho is typically conducted by external research organizations, who employ local staff members. It is important to note the implementing organization’s positive reputation in the district of Butha Buthe due to their longstanding community-based research to address clinical and public health issues in the region (e.g., field research addressing HIV and noncommunicable disease care involving village health workers). In the case of our SWAT, recruiters were Basotho (local ethnicity) who speak Sesotho (local language), who were aware of and used local communication norms to recruit for the host trial. Recruiters for the host trial were employed by the implementing organization for their research roles and moved from other districts approximately 1 year before the SWAT, making them relatively less known among the local community, but still known as research staff from the implementing organization. Future SWATs may test the influence of affiliation disclosure on recruitment rates by randomizing disclosure of the researcher’s affiliation in the recruitment script.

Different dynamics of trust and hope in local healthcare systems versus externally funded health projects (e.g., clinical trials) may also have contributed to the null findings. The study setting is socioeconomically disadvantaged, and the public health system rarely offers innovation for prevalent health issues. Therefore, the desire for health innovation and general improvement of care, including the expansion of treatment options, may supersede the influence of the recruiter’s attire in their willingness to participate in research. This may reflect the instrumental role that external efforts have played in addressing major health challenges in Southern Africa over several generations (e.g., HIV [33]), and how this may have impacted local sentiments toward foreign research in Lesotho. Enrolled participants in the host trial exhibited full understanding that the host trial did not lead to immediate, direct health or monetary benefits through an interviewer-assisted informed consent procedure.

### Limitations

There were several limitations to the SWAT to consider for future studies. Our study team had one male and one female recruiter, making it implausible to generalize on the effect of recruiter gender on participation rates of the survey. Recruiters’ sex differences may play a role in the patients’ attitudes toward a research study, impacting their decision to participate, via gender biases stemming from established gender norms and expectations in the study setting. For example, one could expect that a male recruiter may be more successful in recruiting participants for a study in Lesotho, due to deeply rooted assumptions that males are more socially powerful and competent than females. Contrasting this expectation, descriptive analyses from the current SWAT demonstrate that the recruitment rate of the female recruiter was much higher than the rate achieved by the male recruiter (weighted average recruitment rates—77% vs. 63%, respectively).

Selection biases were also present in this SWAT. By the nature of the in-person recruitment strategies used for the host trial, we only targeted adults who were physically present, waiting at the outpatient clinic, implying that these individuals are more likely to be engaged with public healthcare, and therefore likely to be open to new offers at the health facility (i.e., research study). Seven percent of individuals who did not participate were ineligible by the literacy criterion in this SWAT. It is important to note that literacy is a key structural barrier to survey-based trial participation and may limit the inclusion of key populations in other contexts. In the current study, most nonparticipants cited “time constraint” as a reason to refuse participation, which reflects commonly cited research refusal reasons in other settings [34, 35]. Additionally, we did not record demographic information or visit reasons for all approached potential participants of the host trial (i.e., only recorded for enrolled participants) which limits our understanding of the SWAT’s full sample characteristics. However, this limitation fits within the scope of a SWAT, which is a study design that utilizes host trial’s meta-research data without incurring additional procedures or costs. Finally, the nature of this survey-based, non-invasive behavioral trial may yield different recruitment dynamics than in higher-burden clinical trials (i.e., requiring multiple visits, biospecimens, identifiable information).

Fidelity checks on the recruiter’s adherence to the attire assignment were not conducted systematically. However, several unannounced site checks conducted by the principal investigator did not raise concerns about the recruiter’s adherence to the attire assignments. Finally, the sample size of the host trial was limited, so that the effect estimate of the SWAT is accompanied by a wide confidence interval, not ruling out substantial recruitment increase nor decrease with white coat attire. In future studies testing appearance-based conditions of recruiters, photo confirmation or involvement of a third person to provide in-person oversight may be implemented to ensure adherence to the assigned condition. Follow-up investigations on recruitment determinants may also consider possible behavioral and messaging approaches to increase participation rates in clinical and survey research, such as using framing effects in recruitment scripts.

### Strengths

This methodology is widely applicable to primary data collection being done in almost any in-person recruitment context. This SWAT resulted in little additional cost to the host trial, while providing valid evidence about an important, standalone research question with existing infrastructure and resources. While this SWAT has limitations of only including two recruiters, one man and one woman, such a study can be expanded to include multiple recruiters of the same external demographic characteristics to generate evidence on how a population systematically responds to recruiters of specific age groups or gender.

### Conclusions and implications for future clinical research practice

Optimizing recruitment processes in clinical trials may promote efficiency and ethical practices, particularly in field research involving underserved populations and limited health system resources. While recruiters’ attire did not influence participation rates in Lesotho, there may be other study-side factors that are influential in patients’ decisions to participate in clinical research. Other modifiable recruiter characteristics that may be tested in the future may be age group (i.e., younger versus older recruiters), following the evidence that the provider’s age is influential to patient satisfaction and trust. In terms of the language of the script, future recruitment SWATs can test different behavioral messaging strategies in the scripts used to first approach potential participants. Identifying and delineating these determinants require an in-depth examination of social and medical culture in Lesotho, which has not yet been done. As the current study is the first SWAT to examine determinants of clinical trial recruitment in Lesotho, future SWATs can systematically explore other study-side characteristics, such as different framing techniques and communication modalities, as well as comparisons of recruitment outcomes by the recruiters’ gender and age group in large-scale trials with representative groups of recruiter characteristics.

## Supplementary Information

Supplementary Material 1.

## Figures and Tables

**Figure:**
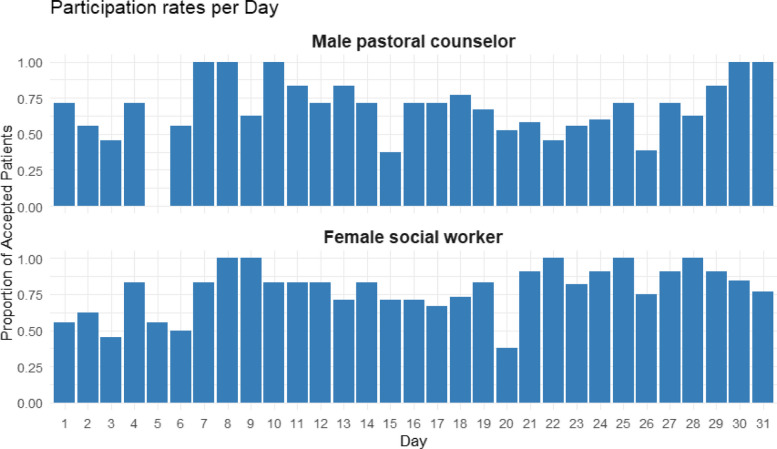
I. Participation rates over time by recruiter

**Figure:**
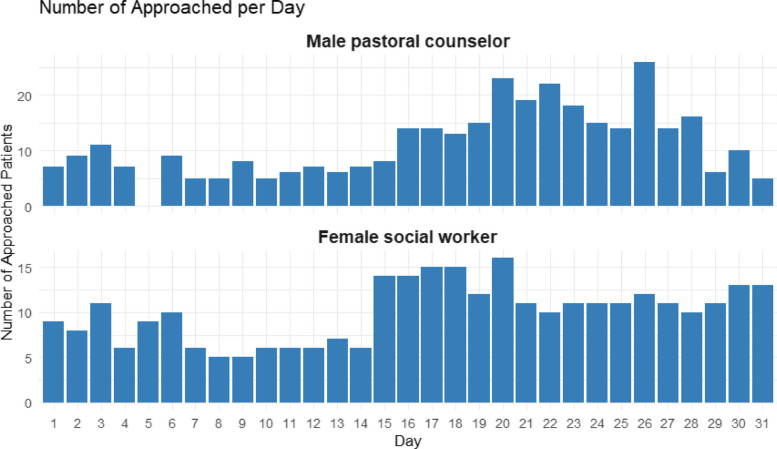
II. Number of approached patients over time by recruiter

**Figure:**
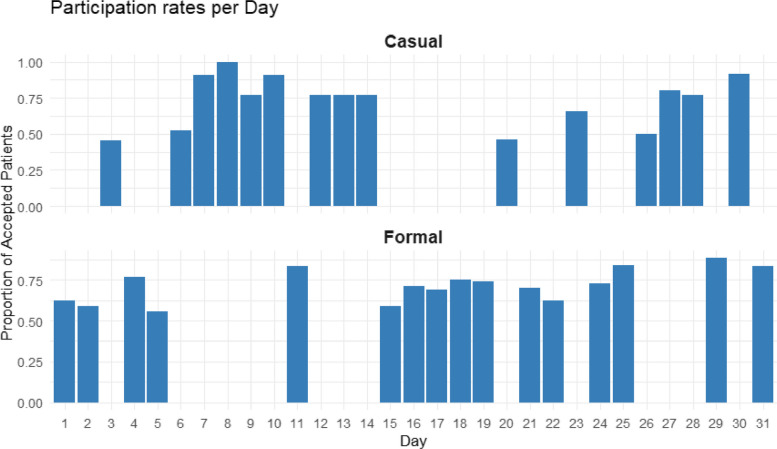
III. Participation rates over time by attire

**Figure:**
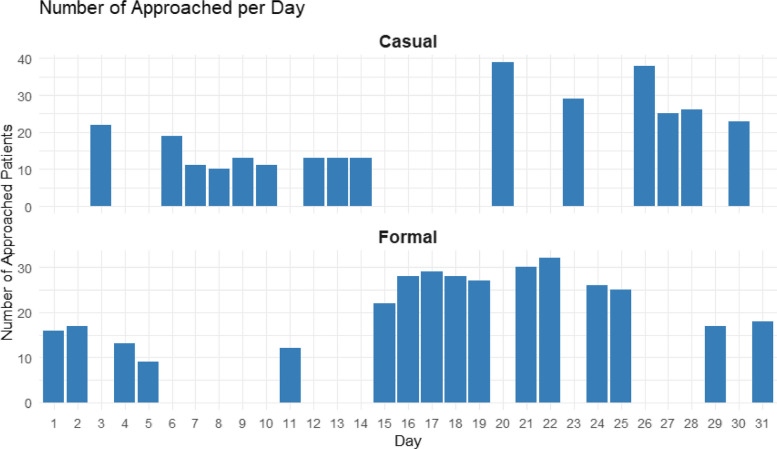
IV. Number of approached patients over time by attire

## Data Availability

The data and code files used for this study are available in the OSF repository (OSF.io/N6xc2).

## Appendix 1

### Interaction effects

Negative binomial mixed model output with interaction term

**Table:**

	Adjusted incidence rate ratio [95% confidence interval]
Attire (Ref: casual)	1.00 [0.77, 1.29]
Recruiter (Ref: female social worker)	0.79 [0.60, 1.04]
Recruiter × attire	1.07 [0.72, 1.53]

## Appendix 2

Sensitivity analyses

I. Participation rates over time by recruiter
II. Number of approached patients over time by recruiter
III. Participation rates over time by attire
IV. Number of approached patients over time by attire

## Abbreviations

(a) IRR: (Adjusted) incidence rate ratio
CI: Confidence interval
HIV: Human immunodeficiency virus
IQR: Interquartile range
LMIC: Low- and middle-income country
LSL: Lesotho loti
STI: Sexually transmitted infection
SWAT: Study within a trial
USD: US dollar
